# Establishment of a hybrid model of atherosclerosis and acute colitis in ApoE^-/^^-^ mice

**DOI:** 10.1371/journal.pone.0289820

**Published:** 2024-03-18

**Authors:** Keke Chen, Shengwei Zhang, Guanghui Cui, Xue Zhang, Yujian Song, Jie Zheng, Yun Chen, Tingting Zheng

**Affiliations:** 1 Shenzhen Key Laboratory for Drug Addiction and Medication Safety, Department of Ultrasound, Institute of Ultrasonic Medicine, Peking University Shenzhen Hospital, The Hong Kong University of Science and Technology Medical Center, Shenzhen- Peking University, Shenzhen, P. R. China; 2 Department of Ultrasound, Nanjing Drum Tower Hospital, Nanjing, P. R. China; 3 Department of Ultrasound, Foshan First People’s Hospital, Foshan, P. R. China; 4 Department of Traditional Chinese Medicine, Peking University Shenzhen Hospital, Shenzhen, P. R. China; Xiangtan University, CHINA

## Abstract

Inflammatory bowel disease (IBD) and atherosclerosis (AS) are both common chronic inflammatory diseases with similar pathophysiological mechanisms. Some studies have shown that IBD patients are at increased risk for early atherosclerosis, myocardial infarction and venous thrombosis. Here we set up a hybrid mouse model associated with atherosclerosis and acute colitis in order to investigate the interplay of the two diseases. We fed ApoE-/- mice with high fat diet to establish atherosclerosis model, and used animal ultrasound machine to detect the artery of mice noninvasively. Then a new hybrid model of atherosclerosis and acute colitis was prepared by drinking water for 7 days. At the end of the experiment, the hybrid model mice showed typically pathological and intuitionistic changes of atherosclerosis and acute colitis. We found the shortened colon length, high histopathological scores of the colon with mucosal erosion and necrosis, hyperlipidemia, a plaque—covered mouse aorta and plaque with foam cells and lipid deposition in the hybrid model group, which proved that the hybrid model was successfully established. At the same time, ultrasonic detection showed that the end-diastolic blood flow velocity and the relative dilation value were decreased, while systolic time / diastolic time, the wall thickness, systolic diameters as well as diastolic diameters were gradually increased, and statistical significance appeared as early as 8 weeks. We clearly described the process of establishing a hybrid model of atherosclerosis and acute colitis, which might provide a repeatable platform for the interaction mechanism exploring and drug screening of atherosclerosis and inflammatory bowel disease in preclinical study.

## Introduction

Despite major advances in medical technology, cardiovascular diseases (CVD) such as ischemic heart disease and stroke remain the leading cause of mortality and disability worldwide. In addition to the recognized traditional cardiovascular risk factors [1, 2], such as smoking, hyperlipidemia, hypertension, diabetes, etc., an increasing number of epidemiological studies have shown that chronic inflammation plays an important role in the pathogenesis of atherosclerosis (AS) and CVD. Many epidemiological studies [3–5] have shown that CVD has been linked to rheumatoid arthritis and systemic lupus erythematosus, however, the association between inflammatory bowel disease (IBD) and CVD remains unclear. Emerging data indicate that the change of intestinal flora in IBD leads to the release of inflammatory cytokines into systemic circulation, thus accelerating the process of AS [6, 7].A recent longitudinal study of 210,162 patients with IBD showed that the 3-month periods before and after inflammatory bowel disease-related hospitalization were associated with an increased risk of acute arterial events in both Crohn’s disease and ulcerative colitis [8]. But other studies [9] have shown that the inflammatory burden in patients with inflammatory bowel disease plays an insignificant role in the development of CVD. Therefore, the preparation of a compound experimental animal model of colitis and atherosclerosis is particularly important in the basic research of disease interaction and the choice of treatment strategies. However, there are currently no concurrent models for these two diseases to study their interaction. We know, the arterial plaque formed by ApoE gene knockout (ApoE−/−) mice fed with high-fat diet is very similar to the pathological morphology and pathological process of human AS, which is a classic model of atherosclerosis. The DSS induced colitis mouse model is simple and reproducible, and can be used to study the pathogenesis of ulcerative colitis (UC) and screen potential therapeutic interventions. Consequently, in this study, we fed ApoE-/- mice with high-fat diet to establish atherosclerosis model for 16 weeks with noninvasive ultrasound of small animals. Then a new hybrid model of atherosclerosis and acute colitis (AC) was prepared by drinking 1.5% DSS water for 7 days. Finally, we verified the successful establishment of the model through serology and pathology.

## Materials and methods

### Animals

8-week-old male ApoE–/– mice (n = 34) and C57BL/6J mice (n = 6) were supplied by the Chinese Academy of Medical Sciences and were raised at the animal laboratory center. The mice were housed in a specific pathogen-free environment with controlled temperature 21±0.5°C, relative humidity of 50%, 12h light/dark cycle and free access to food and water. Then gave them adaptive feeding for 1.5 months. All animal procedures were approved by the Animal Care and Use Committee at Shenzhen PKU-HKUST Medical Center (protocol number 2020–010) and are in accordance with the guidelines of the Animal Experiments of the National Institutes of Health.

### Establishment of hybrid model

ApoE–/– mice were randomly assigned to the HFD group (n = 24) and the ND group (n = 10), and were fed with high-fat diet (RD Western Dirt Company, D12108C) and normal diet (Shenyang Maohua Biotech Co., Ltd) respectively. Six C57BL/6J mice were placed in the wild type (WT) group and were fed with nomal diet. After 16 weeks of continuous feeding, 20 mice were randomly selected from the high fat diet group and then divided into the blank model group (BM, n = 10) and the hybrid model group (DM, n = 10), then, the drinking water of the hybrid model group was changed to 1.5% DSS (MP Biomedicals, cat. no. 160110) water, other conditions were unchanged, and continue feeding for another week [10, 11].

### Ultrasound imaging

At the end of week 0, week 4, week 8, week 12 and week 16, each group of mice were anesthetized by peritoneal injection with 1% pentobarbital sodium (Sinopharm Group Chemical reagent Co., Ltd) at a dose of 50 mg/kg, then after shaving hair from the neck, chest and abdomen carefully with Codex pet electric clipper CP-6800 (Shenzhen Codex Electric Co., Ltd) and depilatory cream (Reckitt Jiahua Co., Ltd), ultrasound transmission gel (Guangdong Guanggong Technology Development Company) is applied to those areas. The Vevo2100 high resolution ultrasonic imaging system for small animals produced by Visualsonics of Canada is used, and the MS440 probe (frequency 30MHz) is selected. First, the probe was placed at the right margin of the mouse sternum and 30° from the median line to obtain the long axis section of the left ventricle. M ultrasound was applied to the proximal ascending aorta above the mouse aortic valve, and the wall thickness (TH), the systolic diameters (Ds) as well as the diastolic diameters (Dd) were measured by the inner edge—inner edge method, and the relative dilation value relD = (Ds-Dd)/Dd was calculated. Secondly, The long axis section of the aortic arch was obtained by placing the probe on the superior right sternum fossa and tilting the probe about 60° to the left, On this section, the innominate artery, the left common carotid artery and the left subclavian artery emerged from the aortic arch in turn. The plaque formation of ascending aorta, aortic arch and innominate artery was observed, and M ultrasound was used to measure TH, Ds, Dd and relD of innominate artery. Third, the probe was placed on the right abdomen to obtain two-dimensional images including the liver and right kidney, and the echo intensity of hepatic and renal cortex was observed to evaluate liver fat content. Finally, place the long axis of the probe slightly to the left of the medial line of the xiphoid process and slide down to the bifurcation of the common iliac artery to observe the long axis section of the abdominal aorta. Pulsed Doppler PW was used to detect the hemodynamic indexes of the abdominal aorta 1cm from the proximal end of the superior mesenteric artery: systolic peak velocity (PSV), end-diastolic blood flow velocity (EDV), resistance index (RI) = (PSV-EDV)/PSV, acceleration time (AT), systolic time (ST), total time (TT) and diastolic time (DT). It should be noted that the angle between the acoustic beam and the blood stream beam in PW mode is less than 60 degrees. The dynamic images of at least 3s are obtained from all sections and saved to the workstation for off-line analysis. The average values of three cardiac cycles are taken for all parameters [12, 13].

### Biochemical analysis

Blood was taken from the tail vein at 0, 4, 8, 12 and 16weeks to measure the total cholesterol (TCH) and blood sugar (GLU) levels by BeneCheck multi-function analyzer (Qinli Biotechnology Co., Ltd). And we read the value directly according to the default setting of the instrument.

### Atherosclerotic lesions analysis

We killed the mice by isoflurane anesthesia (Forene, Abbot, cat. no. 4831850) plus cervical dislocation. After blunt dissection of peripheral adipocyte tissue of mouse aorta with pliers, the whole length of aorta was cut to bilateral common iliac artery for Sudan IV (Sloarbio Corporation) staining, and then Image-pro Plus 6.0 software was used to analyze and quantify the red-stained area. The aortic roots were fixed with 4% paraformaldehyde solution (Biosharp Corporation), embedded in paraffin (Leica Corporation), sectioned, and finally stained with hematoxylin and eosin (Biosharp Corporation).

### Pathological analysis of colon, cecum, heart, liver and kidney

After euthanasia of the mice, we opened the mice abdominal cavity and tookout the whole colon and rectum from anus to ileocecum then measure its length. At the same time, we removed the heart, liver and kidney of mice. The above tissues were fixed by formaldehyde, embedded in paraffin, sliced and stained by HE. The images were observed under a light microscope (OLYMPUS, U-LHEAD, Japan) and evaluated in a blinded fashion by an experienced pathologist. The severity of colonic injuries was graded semi-quantitatively according to previous methods by two investigators who were blinded to the grouping (S1 Table) [14, 15].

### Statistical analysis

GraphPad Prism 5.0 software (GraphPad Software Inc., San Diego, CA, USA) was used to analyze the data. Results are expressed as mean ± standard error of the mean (SEM) from at least 3 independent experiments. Statistical significance between two groups was analyzed using the Student’s t-test. Differences among three or more groups were evaluated using one-way ANOVA, followed by Duncan’s multiple range tests. Differences were considered significant at P < 0.05.

## Results

### Ultrasonographic findings of atherosclerosis

**Fig 1:**
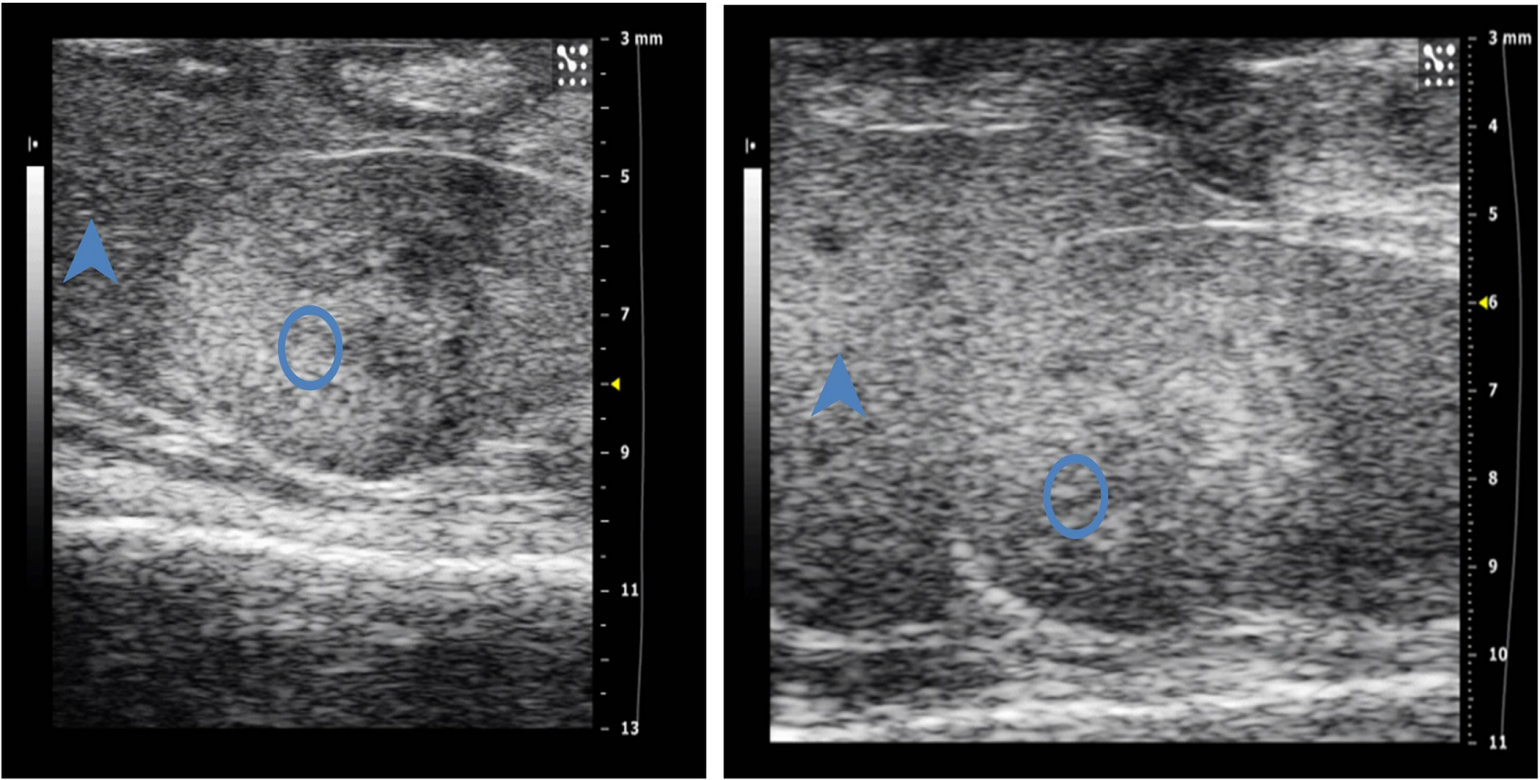
Two-dimensional gray scale ultrasound of liver and right kidney in mice at 16 weeks. **1.** The left image shows the liver and kidney images of ND group, the right image shows the liver and kidney images of HFD group. (The arrow points to the ultrasound image of the liver, and the oval is the kidney).

As shown in S2 and S3 Figs, the TH, Ds and Dd of ascending aorta and innominate artery gradually increased, and the relD value decreased gradually, and there was a significant difference between HFD group and ND group at 8 and 12 weeks separately. As shown in S4 Fig, the EDV of abdominal aorta decreased gradually with the prolongation of feeding time, and there was a significant difference between HFD group and ND group from the 8 weeks, and the difference was the most significant at the 12 weeks. The ST/DT, RI and AT/TT of abdominal aorta increased gradually, but there was no statistical difference between the latter two groups and ND group, while the former group had a statistical difference at 12 weeks. In addition, the PSV of abdominal aorta did not change significantly and there was no statistical difference between the ND group (S5 Fig). At the end of 16th week, gray scale ultrasound was used to observe the echoes of the liver and right kidney of mice. The results showed that the echoes of the right kidney cortex of mice in the ND group were much higher than those of liver parenchyma, while the echoes of the liver in 14 out of 20 surviving mice in the HFD group was diffusely enhanced, higher than that of the kidney cortex, showing fatty liver image. The sensitivity of two-dimensional ultrasound in the diagnosis of mouse fatty liver was 70% (Fig 1).

### Serological indicator

**Fig 2:**
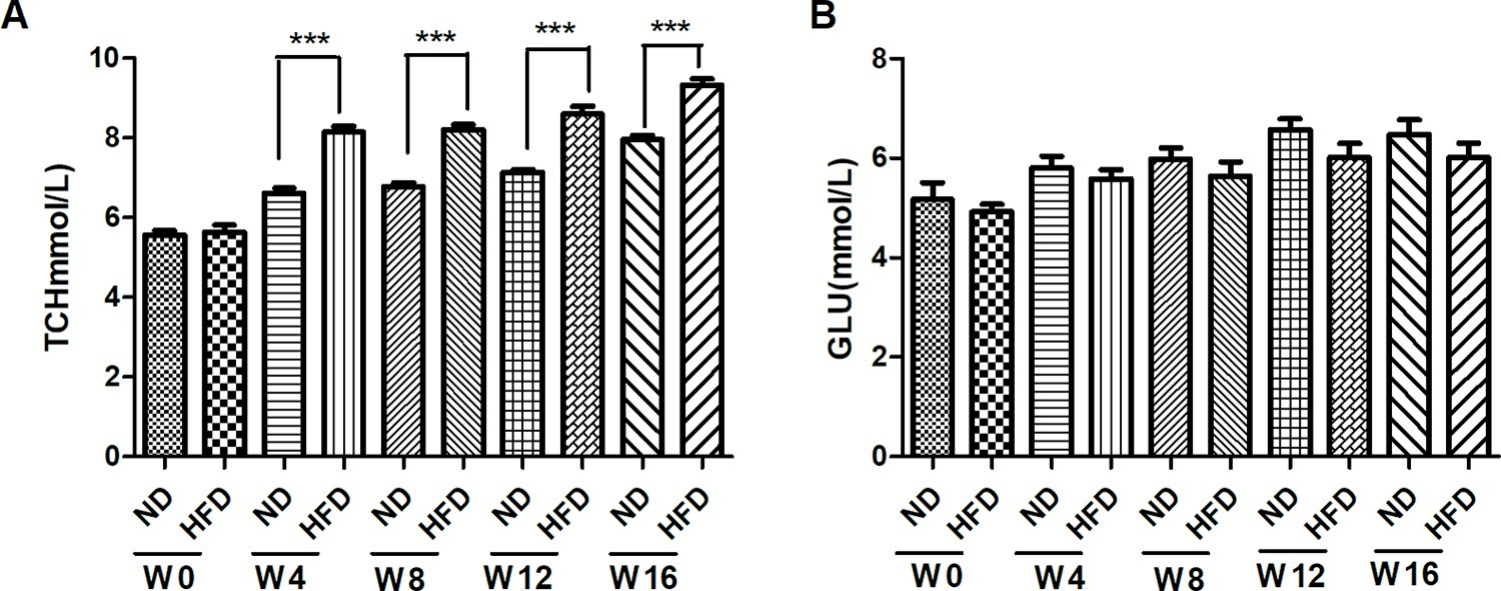
The serological indicator in ApoE–/–mice. **2.** (A) Total cholesterol level, (B) Blood glucose level. Data are expressed as mean ± SEM. Statistical significance was determined by t-test. ***p< 0.001, vs ND.

Total cholesterol levels in the HFD group were higher than those in the ND group, which was statistically significant starting at week 4. However, there was no significant difference in blood glucose levels between the two groups at all stages (Fig 2).

### The gross and pathological manifestations of atherosclerotic lesions

**Fig 3:**
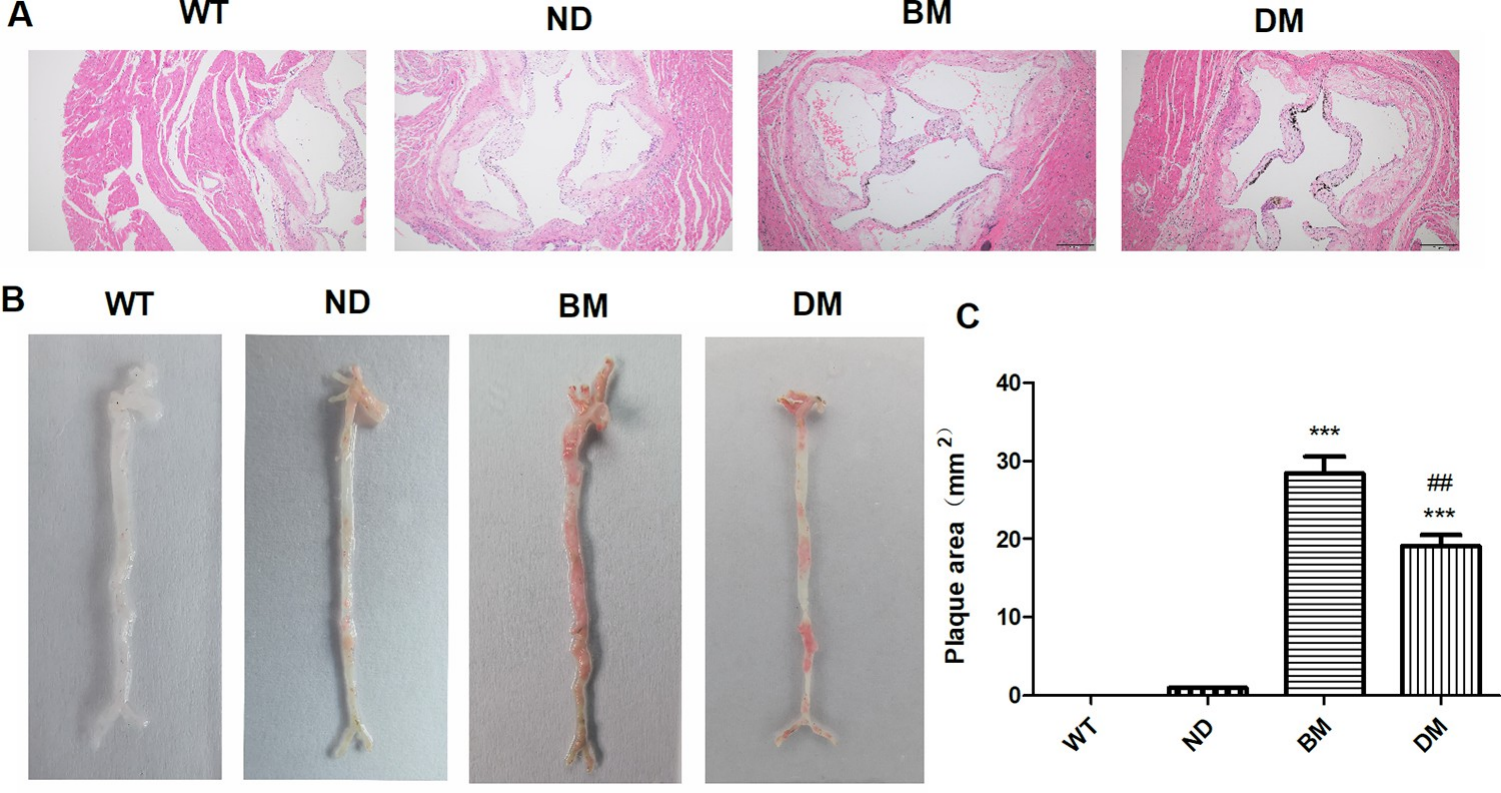
The degree of atherosclerotic lesions. (A) HE staining of aortic root. (B) Representative images of Oil Red O staining of aorta. (C) Quantitative analysis of plaque area in aorta. Data are expressed as mean ± SEM. Statistical significance was determined by t-test. ***p< 0.001, vs ND; ###p< 0.001, vs BM.

The intima of the aorta of mice in BM group and DM group was significantly thickened, with a large number of lipids, necrotic matter, foam cells and calcification focus. Smooth muscle cells in the media were compressed and atrophied, elastic fibers were destroyed, and lymphocyte infiltration was observed in the outer membrane. The above changes were also observed in the aorta of mice in ND group, but the pathological changes were less serious than those in the model group. And no abnormal pathological findings were found in the intima aorta of WT mice (Fig 3A). The gross specimens of the aorta in BM group and DM group showed large amounts of plaque formation everywhere, among which the abdominal aorta was the heaviest, followed by the ascending aorta, and the thoracic aorta was the lightest, and the formation of abdominal aortic aneurysm was also observed in some arteries. However, only a small number of red-stained areas were observed throughout the aorta of ND mice, mainly abdominal aorta and ascending aorta, and no plaque was observed in the aorta of WT group (Fig 3B). According to statistical analysis of plaque area, the plaque area of BM group and DM group was significantly larger than that of ND group, while that of DM group was smaller than that of BM group, with statistical significance (Fig 3C). These data suggest that HFD feeding significantly promotes the development of atherosclerosis while DSS can improve HFD-induced atherosclerosis.

### HE staining of the liver, kidney and heart

**Fig 4:**
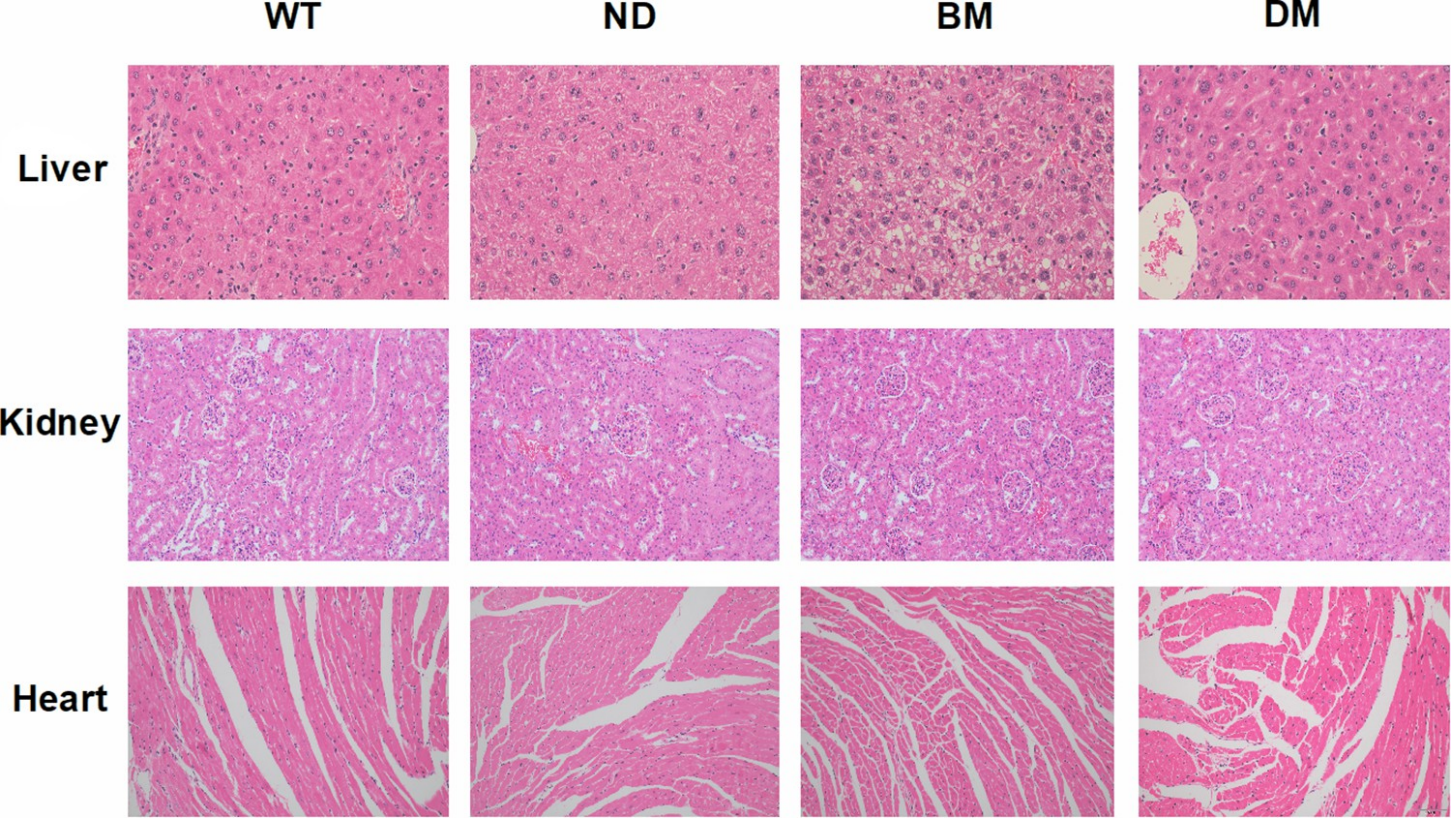
HE staining of liver, kidney and heart in mice of different ages (magnification, ×400, ×200, ×200).

Vesicular steatosis was observed in the liver tissue of BM group, while no fat vacuole was found in the WT, ND and DM groups. In addition, neutrophil infiltration was observed around the vein of hepatic portal area in DM group. There were no abnormal changes in the morphology and structure of kidney and heart tissue and no inflammatory cell infiltration in the four groups (Fig 4).

### Morphology and histology of colon and cecum

**Fig 5:**
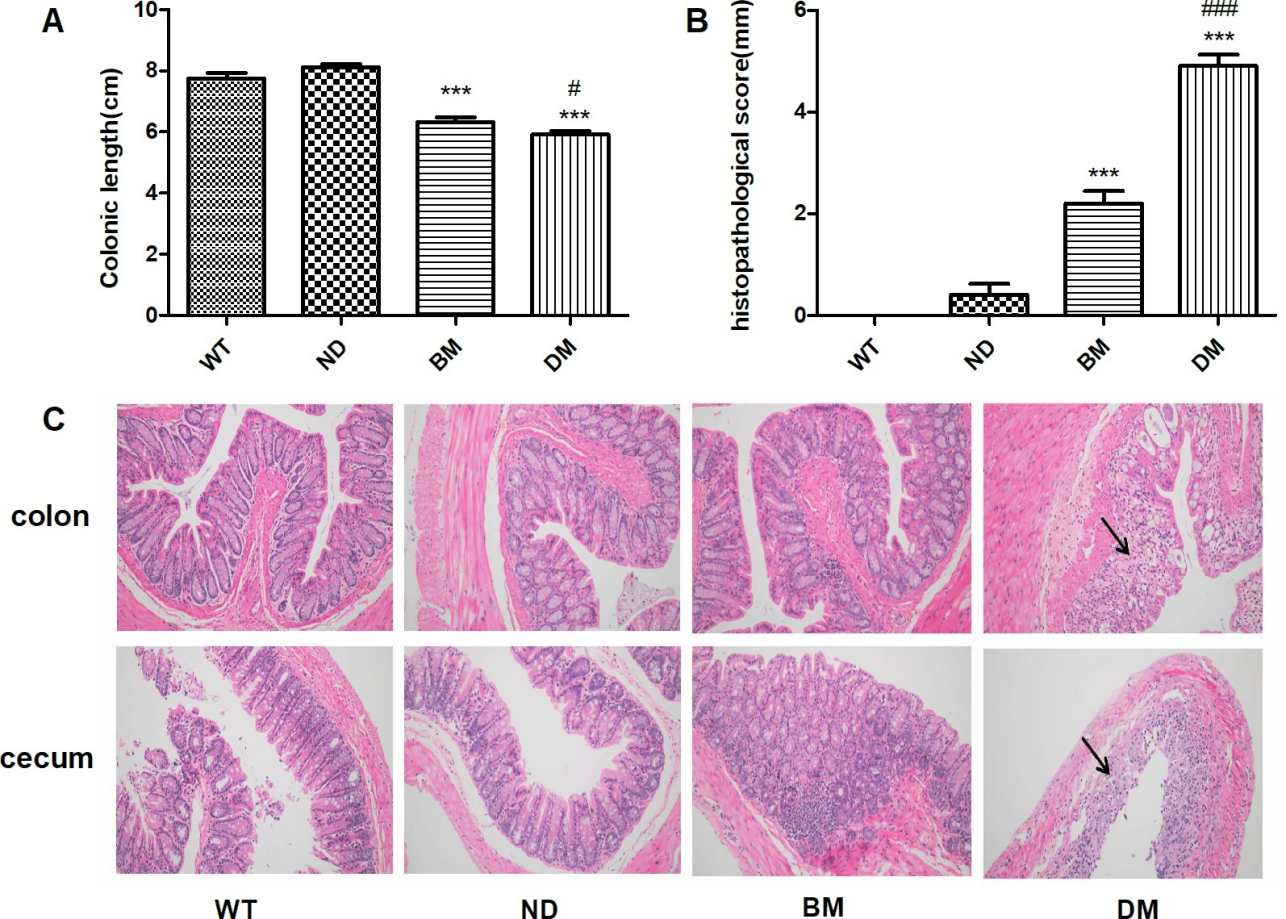
Morphology and histology of colon and cecum. **5.** (A) The colon length of mice. (B) Histopathological score of the colon. (C) HE staining of colon and cecum (magnification, ×100), the arrow points to a collection of neutrophils. Data are expressed as mean ± SEM. Statistical significance was determined by t-test. ***p< 0.001, vs ND; #p< 0.05, vs BM.

Fig 5A showed that compared with ND group, the colon of BM group and DM group was significantly shorter (P < 0.001), especially in DM group, but there was no significant difference between ND group and WT group. The histopathological score of the colon in DM group (4.90±0.23) was significantly higher than that of BM group (2.20±0.25) and the other two groups (Fig 5B). HE staining (Fig 5C) showed a strong inflammatory response at the end of the colon in the DM group, including mucosal erosion, necrosis, thinning, submucosal congestion, edema, and extensive neutrophil infiltration. While the thickness of cecum and colon mucosa in WT, ND and BM groups was uniform and complete without ulcer and inflammatory cell infiltration, and the structure of crypt was normal.

## Discussion

In this study, we fed ApoE-/- mice with high fat diet to establish atherosclerosis model, and used Vevo2100 small animal ultrasound machine and ultra-high resolution MS-400 vascular probe to detect the relevant parameters of the artery. At the end of the experiment, the Sudan IV staining of the aorta in the BM group showed a large number of plaques, mainly located in the aortic arch and abdominal aorta, and the plaque area was larger than that in the WT and ND groups. The difference was statistically significant (P <0.05). HE staining of liver in BM group showed vacuolar degeneration, indicating the formation of fatty liver. HE staining of the aortic root in the BM group showed significant thickening of the intima of the artery, a large amount of lipid deposition, and a large number of inflammatory cells. In summary, we finally confirmed the successful establishment of mice atherosclerosis model. We know that after plaque rupture, the coagulation cascade reaction can be activated, resulting in thrombin production, platelet activation and thrombotic events. Some studies [16] have shown that the plaque of AS model in ApoE-/- mice is not easy to rupture, so it is impossible to simulate acute myocardial infarction and stroke caused by plaque rupture in clinic. However, we found for the first time in HE stained sections of the aortic root that there were aggregates of hemosiderin and flaky fresh red blood cells in the intima of the blood vessels, which indicated that there were old and fresh capillary rupture bleeding in the plaque, that is, rupture bleeding in the plaque was persistent. Therefore, we speculated that acute coronary syndrome may have occurred in mice in the BM group, which also indicated that the modeling of this model was very rapid and severe.

In the ultrasonic data, we found that with the extension of feeding time and the aggravation of atherosclerosis, the TH of ascending aorta and innominate artery in ND and HFD groups increases gradually, and the relD decreases gradually, which suggests that besides the classical index of atherosclerosis, the dilatation of arteries and the decrease of relD may also be an early indicator of atherosclerosis. Related studies [17] have also confirmed that atherosclerosis can cause vascular positive remodeling and arterial dilatation can be used as an early marker for the detection of vascular diseases. In addition, compared with ND group, the ascending aorta of HFD group first showed statistical difference at 8 weeks, and the innominate artery had statistical difference at 12 weeks, so it can be inferred that the development direction of atherosclerosis is from near to far. Finally, we measured the PSV, EDV, RI, ST/DT, AT/TT of the abdominal aorta and found that EDV showed a decreasing trend. It is well known that diastolic blood flow is caused by elastic retraction of the great arteries, so the continued decline of EDV also reflected the decreased compliance of vessel walls caused by atherosclerosis. In order to eliminate the effect of heart rate differences caused by self-factors and anesthetic factors on the results, we measured the AT/TT of a cardiac cycle and ST/DT to measure the proportion of AT, ST and DT in the whole cardiac cycle. The ST/DT of the HFD group increased continuously, and there was a statistical difference compared with the control group at 12 weeks, and the AT/TT also showed an increasing trend, but there was no significant difference between the two groups. These changes may be related to increased vascular resistance and more strenuous contraction. The same results showed that the ST increased with the development of atherosclerosis [18]. In this study, it was also observed that the RI of the two groups showed an increasing trend, but there was no significant difference between the HFD group and the ND group, which may be related to the ApoE-/- mice in the ND group which would also progress to AS. Another interesting finding is that with the extension of feeding time, some mice in the HFD group showed disappearance or even reversal of the diastolic phase of the abdominal aorta on color doppler. Some mice in the HFD group had ascending aortic valve regurgitation, which may be caused by the weakening of the elasticity of the ascending aorta, the dilatation of the lumen, and the relative insufficiency of the aortic valve. This phenomenon can be verified by increasing the sample size and prolonging the modeling period in the follow-up study. Therefore, the above results show that compared with traditional autopsy, ultrasound can be used for non-invasive, accurate and dynamic evaluation of atherosclerosis in ApoE-/- mice.

Then, the hybrid model established on the basis of atherosclerosis model using a concentration of 1.5% DSS [19–21]. On the second day after the initiation of DSS, the mice began to show the typical characteristics of colitis, which was characterized by sparse stool, a small amount of bleeding in feces, and obvious bleeding on the 4th day. At the end of the experiment, the colons of mice in DM and BM group were significantly shorter than those in ND group (P<0.05), especially in DM group. HE staining of the colon in DM group showed obvious inflammatory changes such as goblet cell loss, crypt destruction and lymphocyte infiltration. The histopathological score was significantly higher than that in the other three groups. In addition, neutrophil granulocytes were observed on the HE staining of the liver in DM group, which may be related to the infiltration of enteritis. The results of this study showed that AC model could be successfully established in mice treated with 1.5%DSS for one week. In addition, we found that no adipose vacuole was observed in liver sections of the DM group after HE staining, and the plaque area of mice in the DM group was less than that in the BM group after Sudan IV staining, with statistical significance (P<0.05). This suggests that acute enteritis may have a reverse effect on atherosclerotic plaque, which is an additional interesting finding that seems to provide new insights into the treatment of atherosclerosis.

## Conclusion

In summary, twelve weeks of high-fat diet combined with one week of 1.5%DSS water can quickly and successfully establish the AS-IBD hybrid model with the noninvasive monitoring of ultrasound. Mold can provide a repeatable platform for the study of the pathogenesis and the evaluation of therapeutic efficacy of AS and IBD.

## Supporting information

S1 Fig Ultrasound images of blood vessels in mice. (TIF)

S2 Fig Ultrasonic measurements of the ascending aorta in ApoE-/- mice. (TIF)

S3 Fig Ultrasonic measurements of the innominate artery in ApoE-/- mice. (TIF)

S4 Fig Ultrasonic measurements of the abdominal aorta in ApoE-/- mice. (TIF)

S5 Fig The end- systolic velocity of abdominal aorta. (TIF)

S6 Fig Colonic length. (TIF)

S7 Fig Composition of intestinal flora. (TIF)

S1 Table Histological scoring criteria for the colon. (TIF)
