# Supplementary figures and images for: Establishment of a hybrid model of atherosclerosis and acute colitis in ApoE-/- mice
Source: PLoS One. 2024 Mar 18;19(3):e0289820. doi: 10.1371/journal.pone.0289820 (PMC10947657; doi:10.1371/journal.pone.0289820)

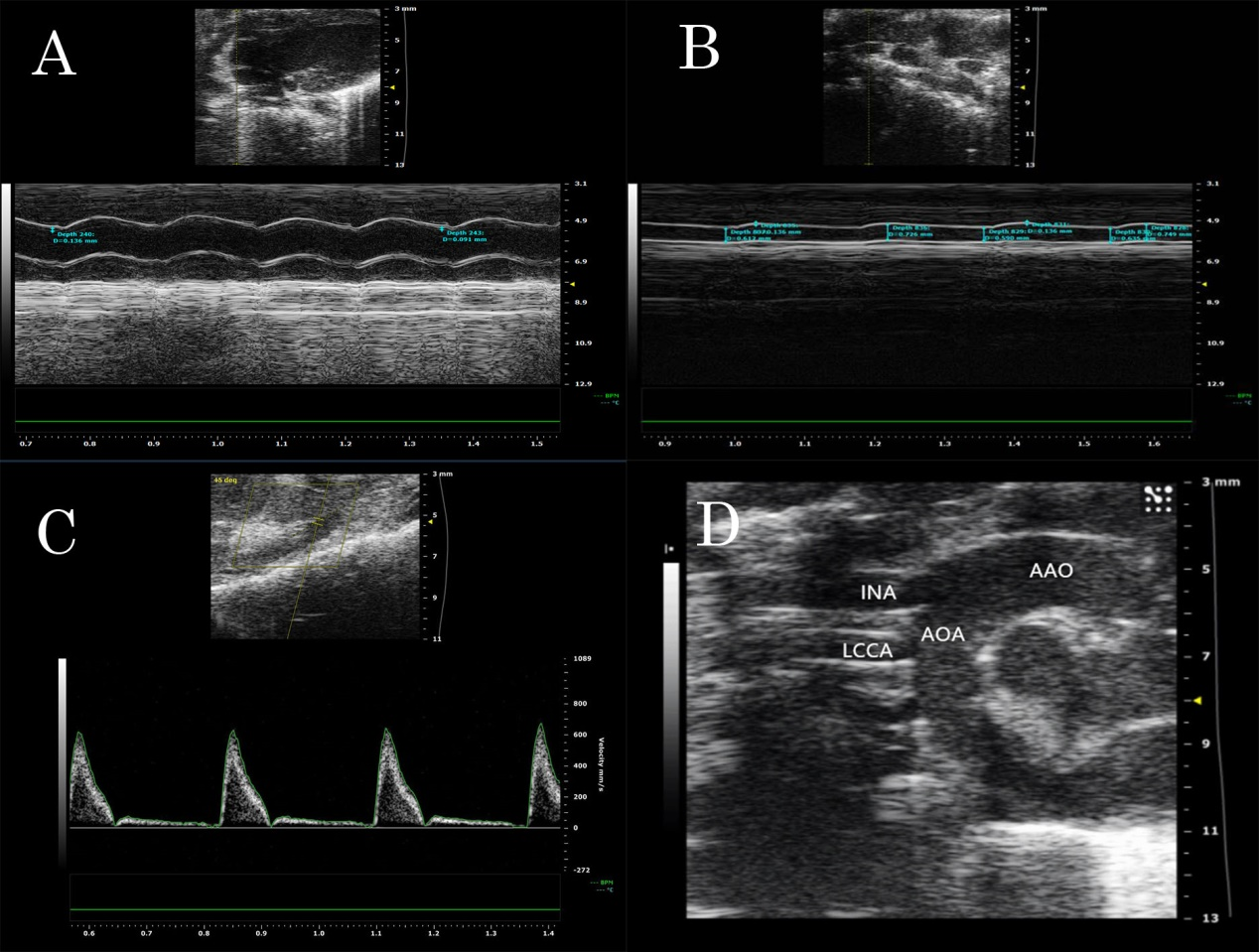

Supplement: S1 Fig — (TIF) [file pone.0289820.s001.tif]

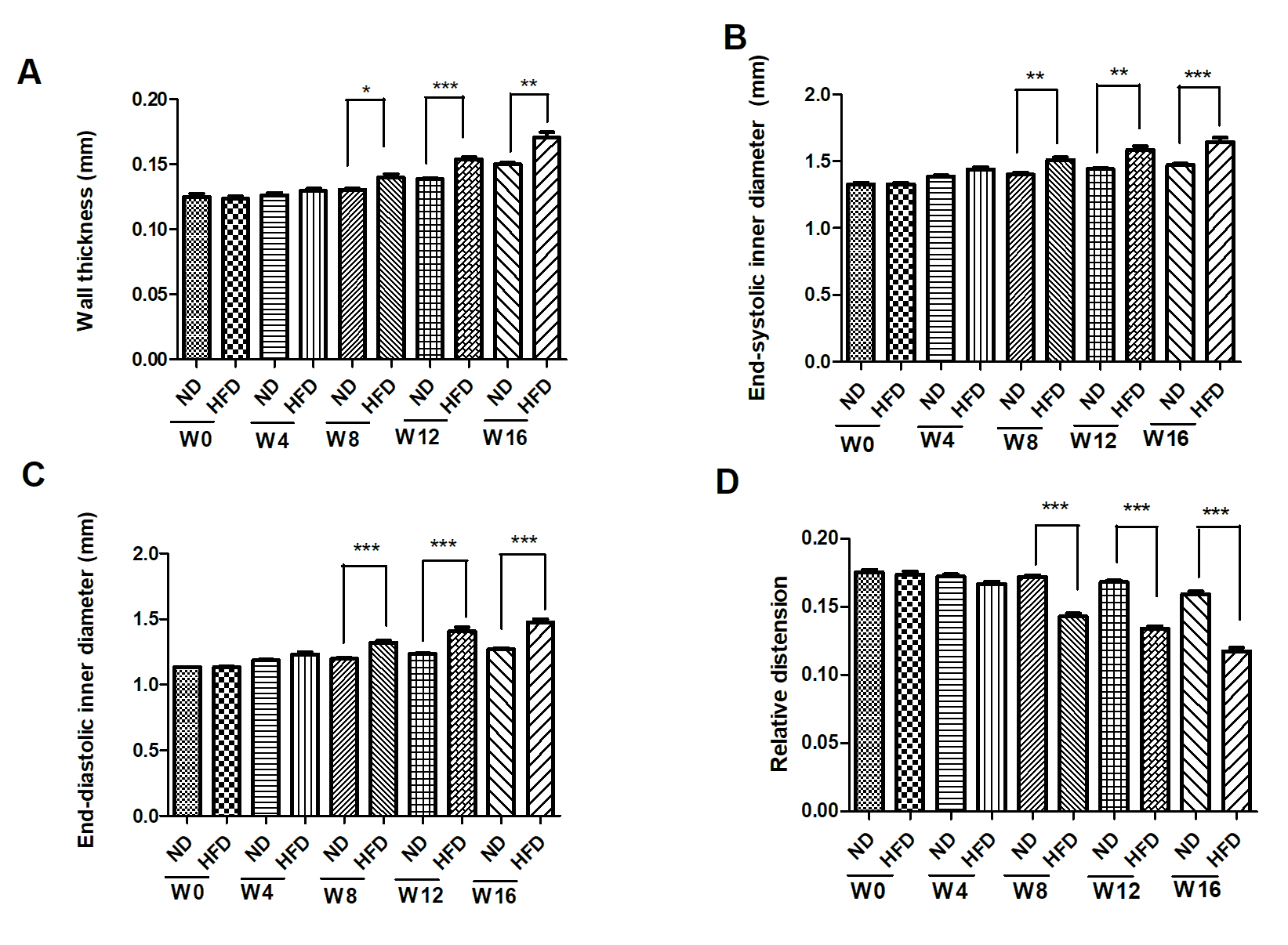

Supplement: S2 Fig — (TIF) [file pone.0289820.s002.tif]

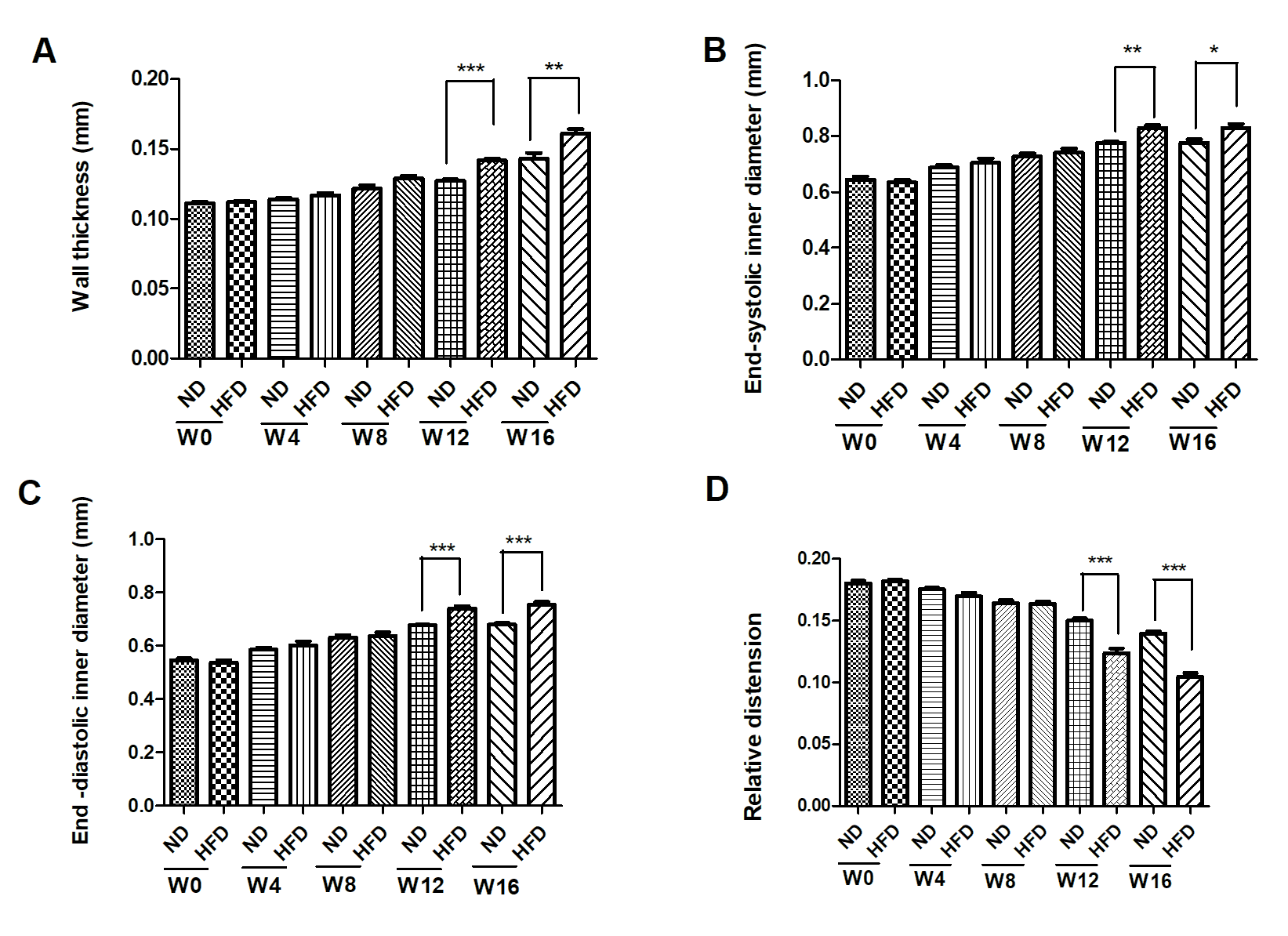

Supplement: S3 Fig — (TIF) [file pone.0289820.s003.tif]

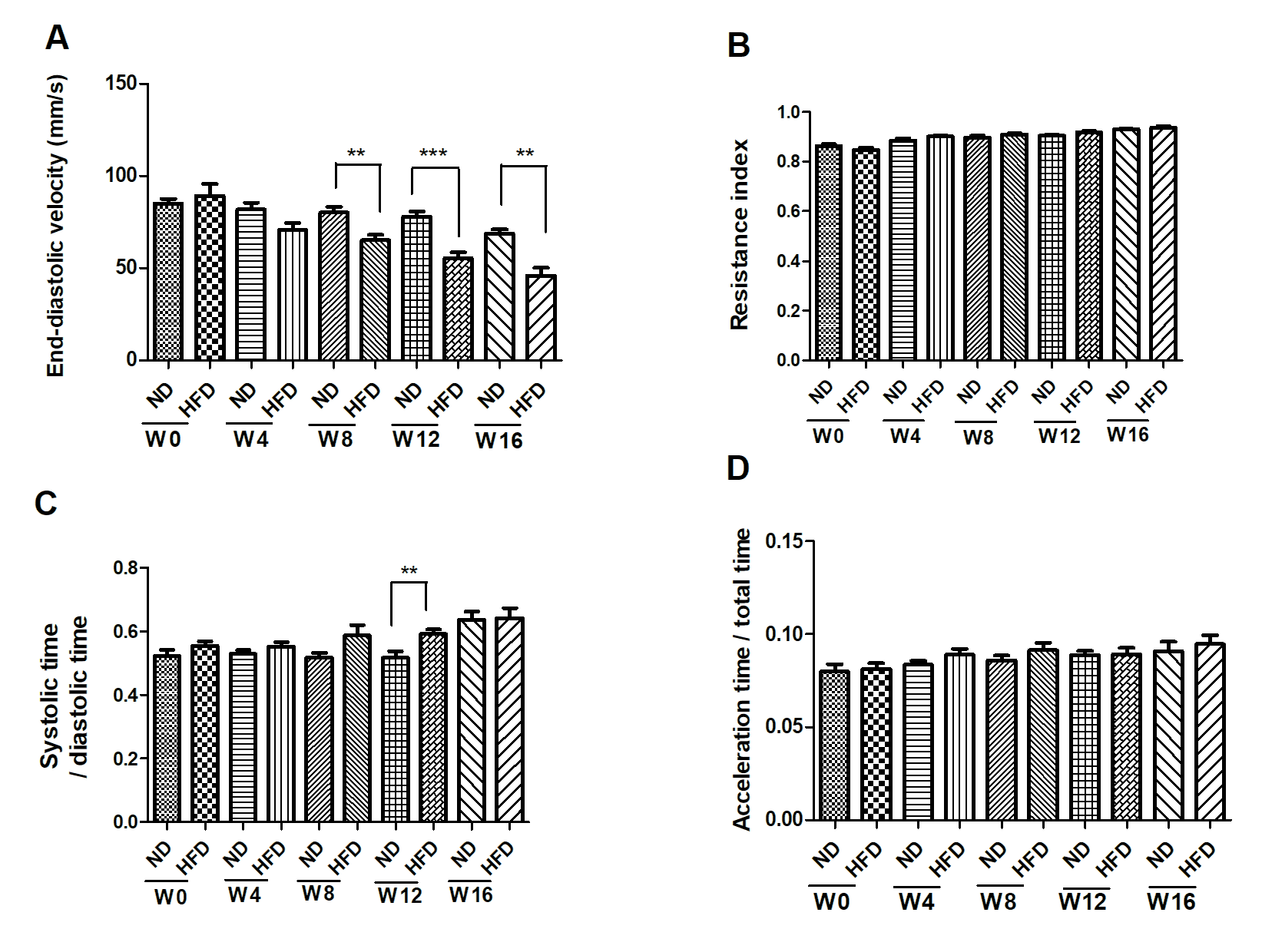

Supplement: S4 Fig — (TIF) [file pone.0289820.s004.tif]

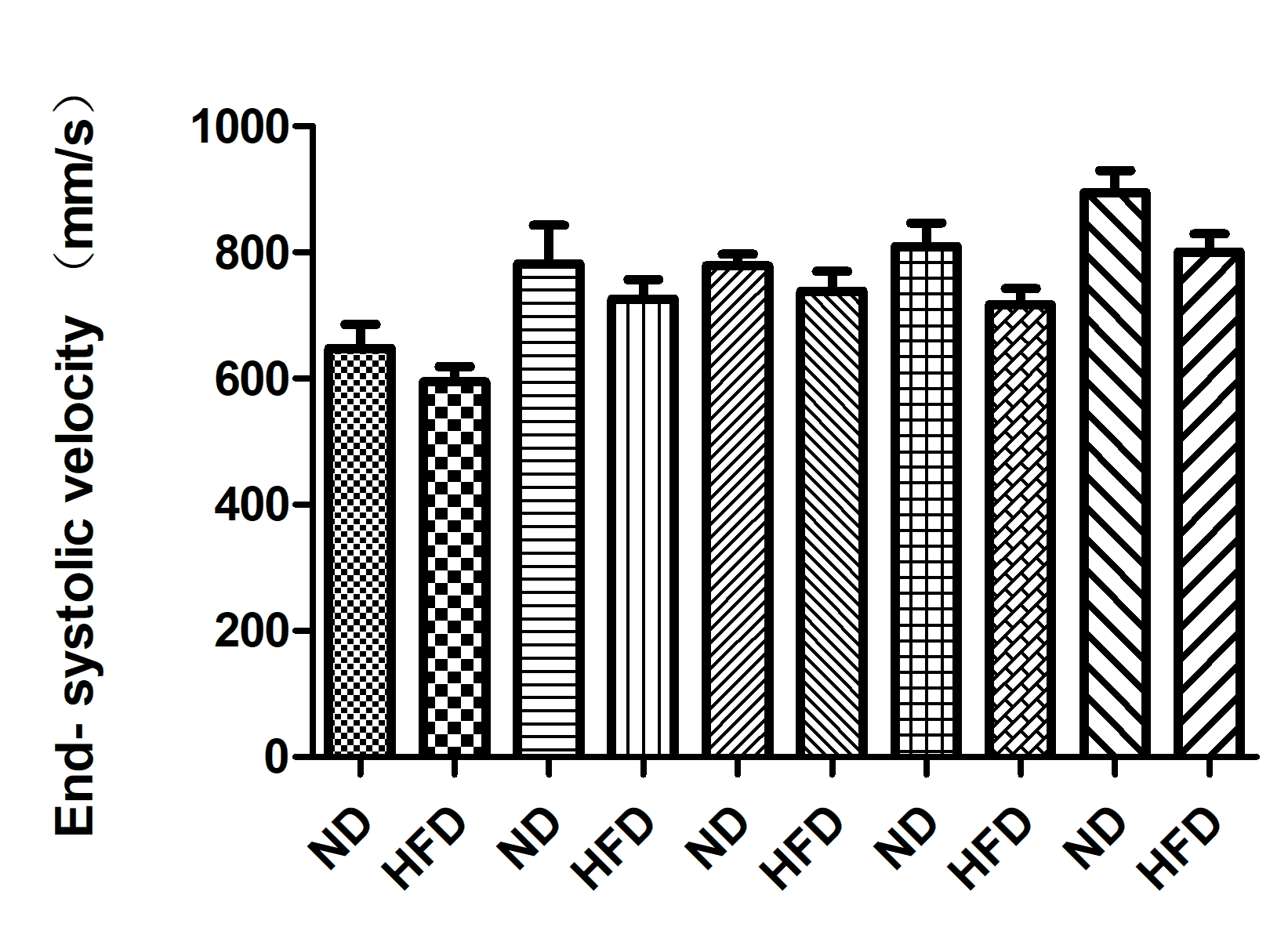

Supplement: S5 Fig — (TIF) [file pone.0289820.s005.tif]

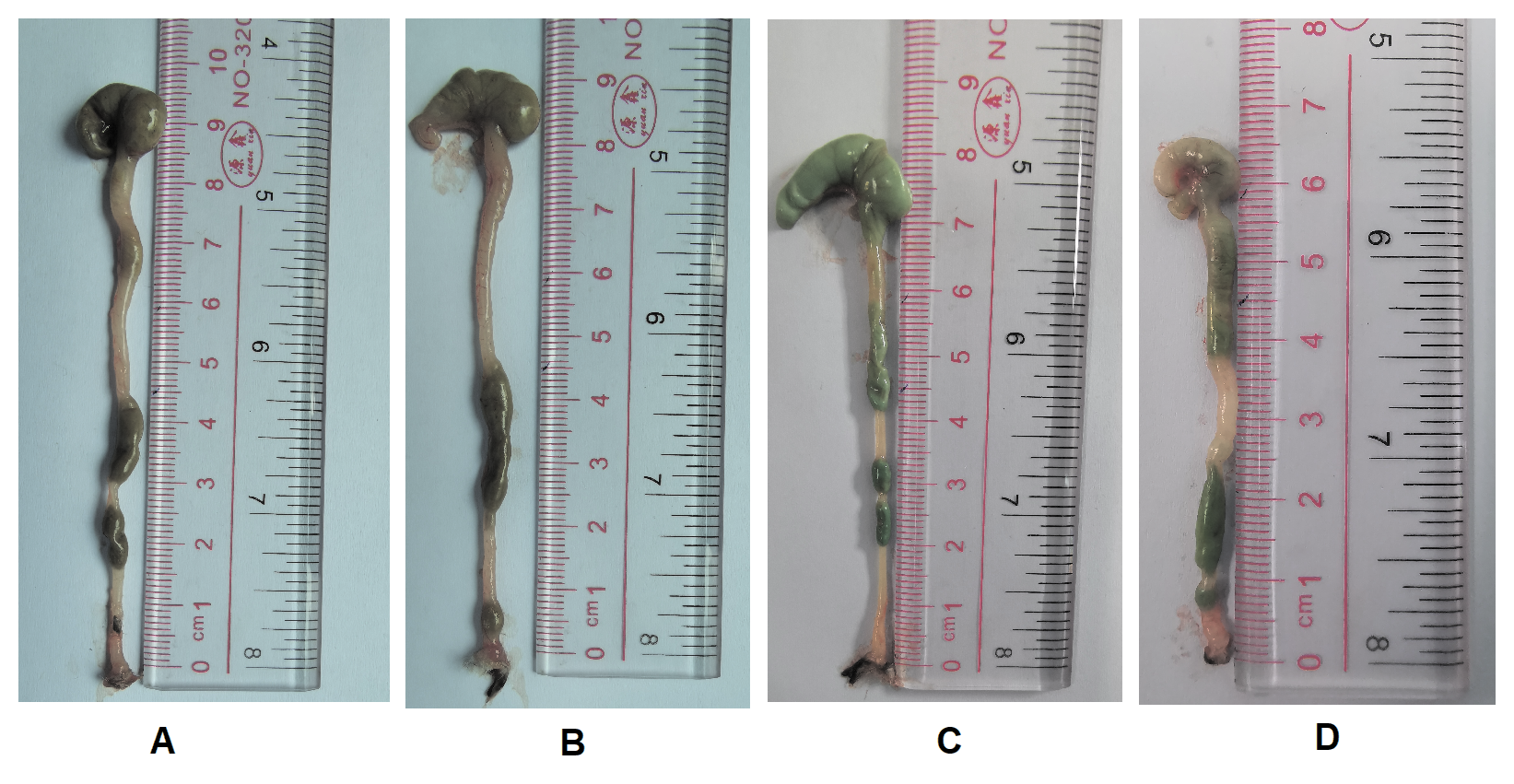

Supplement: S6 Fig — (TIF) [file pone.0289820.s006.tif]

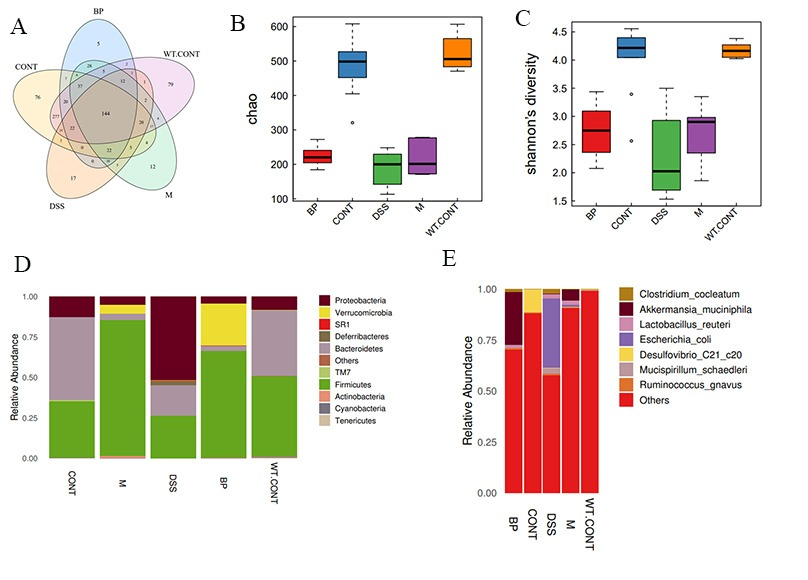

Supplement: S7 Fig — (TIF) [file pone.0289820.s007.tif]

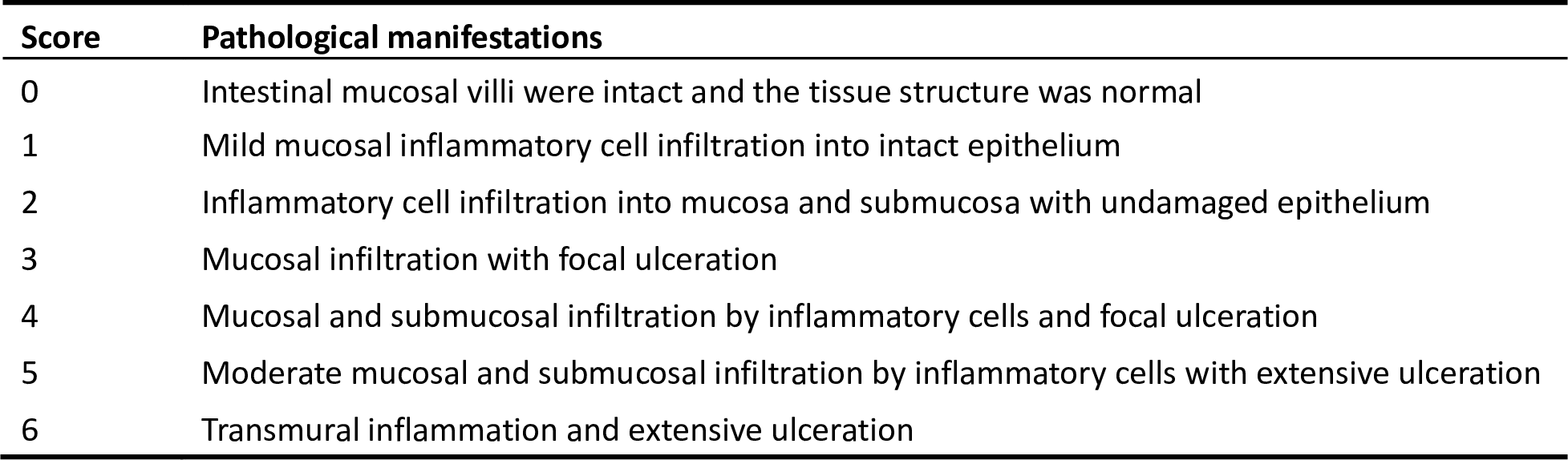

Supplement: S1 Table — (TIF) [file pone.0289820.s008.tif]
